# KSHV SOX mediated host shutoff: the molecular mechanism underlying mRNA transcript processing

**DOI:** 10.1093/nar/gkw1340

**Published:** 2017-01-28

**Authors:** Hyunah Lee, Anathe O.M. Patschull, Claire Bagnéris, Hannah Ryan, Christopher M. Sanderson, Bahram Ebrahimi, Irene Nobeli, Tracey E. Barrett

**Affiliations:** 1 Institute for Structural and Molecular Biology, Department of Biological Sciences, Birkbeck College, Malet Street, London WC1E 7HX, UK; 2 Department of Structural and Molecular Biology, University College London, London, WC1E 6BT, UK; 3 Liverpool School of Tropical Medicine, Pembroke Place, Liverpool, L3 5QA, UK; 4 Department of Cellular and Molecular Physiology, Institute of Translational Medicine, University of Liverpool, Crown Street, Liverpool, L69 3BX, UK; 5 Department of Functional and Comparative Genomics, Institute of Integrative Biology, University of Liverpool, Crown Street, Liverpool L69 7ZB, UK

## Abstract

Onset of the lytic phase in the KSHV life cycle is accompanied by the rapid, global degradation of host (and viral) mRNA transcripts in a process termed host shutoff. Key to this destruction is the virally encoded alkaline exonuclease SOX. While SOX has been shown to possess an intrinsic RNase activity and a potential consensus sequence for endonucleolytic cleavage identified, the structures of the RNA substrates targeted remained unclear. Based on an analysis of three reported target transcripts, we were able to identify common structures and confirm that these are indeed degraded by SOX *in vitro* as well as predict the presence of such elements in the KSHV pre-microRNA transcript K12-2. From these studies, we were able to determine the crystal structure of SOX productively bound to a 31 nucleotide K12-2 fragment. This complex not only reveals the structural determinants required for RNA recognition and degradation but, together with biochemical and biophysical studies, reveals distinct roles for residues implicated in host shutoff. Our results further confirm that SOX and the host exoribonuclease Xrn1 act in concert to elicit the rapid degradation of mRNA substrates observed *in vivo*, and that the activities of the two ribonucleases are co-ordinated.

## INTRODUCTION

KSHV predominantly targets immunocompromised individuals and has been directly linked to Kaposi's sarcoma (KS), the most common form of AIDS-related cancer together with lymphoproliferative disorders that include multicentric Castleman's disease and primary effusion lymphoma (PEL) (1). In common with members of the γ-herpesviridiae, KSHV has a biphasic life cycle and following an as-yet-unknown stimulus, can transition from the latent to lytic phase. This is concomitant with the large-scale overexpression of viral genes required for genomic replication and the ultimate production of progeny. Transition to the lytic phase coincides with the rapid degradation of ∼80% of all host mRNA transcripts in a process termed host shutoff (HSO) that is thought to promote immune evasion and enable viral co-option of the host replicative machinery (2). In the γ-herpesviridiae, HSO is initiated by the bifunctional alkaline exonuclease SOX that has been shown to possess exonucleolytic DNase and RNase activities (3,4). Owing to the presence of a PD-(D/E)XK sequence spanning two of seven conserved motifs shown to be essential for DNA turnover, KSHV SOX has been classified as a member of the type II restriction endonuclease-like superfamily (5) confirmed by the crystal structures of the the related Epstein–Barr virus alkaline exonuclease BGLF5 (3) and apo form (6). In addition, a bridge or arch structure linking the N and C-terminal lobes of SOX is also present (similar to that observed in bacteriophage lambda exonuclease) that has been shown to function in substrate recognition in flap endonucleases and suggested to have a similar role by mutagenesis studies performed in BGLF5 (3,6–8). The crystal structure of SOX bound to a DNA duplex has been reported which revealed that the degradation of DNA substrates is likely to proceed via a bi-metal nuclease mechanism involving the catalytic carboxylate groups D221 and E244. Although it has been shown that the processing of DNA and RNA substrates requires the same catalytic center (4,9), a number of residues have been identified that attenuate HSO while having no impact on DNA processing. The exact manner in which they participate, however, is unclear given their disparate nature, broad spatial distributions and the fact that none were observed to impair SOX’ 5΄-3΄ exonucleolytic RNase activity (10).

Despite the *in vitro* detection of a 5΄-3΄ exonucleolytic RNase activity against unstructured oligonucleotides, it was unclear whether they were the only targets (4). More recent *in vivo* studies, however, indicate that SOX-mediated HSO is instead initiated by endonucleolytic ‘nicking’ of target transcripts followed by exonucleolytic degradation by the co-opted host scavenger exoribonuclease Xrn1 reminiscent of nonsense-mediated decay (9). It has also been reported that a pre-requisite for processing is a degenerate sequence containing two or more unpaired adenine nucleotides located 5΄ to the incision site within the context of a stem loop structure (11). Furthermore, cleavage has been cited to preferentially occur directly 5΄ to pyrimidines or adenine where guanine is highly under-represented. Despite these advances, it has yet to be established whether specific elements or more general characteristics are being recognized for endonucleolytic mRNA processing given that a number of the targets lacked some or all of these features. In this study, common stem loops and bulges were identified in a detailed *in silico* analysis of three distinct mRNA reporter transcripts shown to be degraded by SOX *in vivo* (9). Based on our findings, we were able to both predict and verify the presence of similar elements in the KSHV pre-microRNA (pre-mirna) K12-2 (K2), an important factor in the suppression of anoikis (12). A 31mer K12-2 fragment was subsequently co-crystallized with a catalytically inactive SOX mutant to yield a productive complex. This first structure of a viral pre-miRNA fragment bound to a viral nuclease in conjunction with biochemical/biophysical studies reveals that despite targeting stem loop elements and bulges, SOX-mediated turnover appears not to require recognition of a particular consensus sequence and that there are no obvious restrictions on the size of the loop/bulge elements incised. Through re-evaluating five non-catalytic HSO mutants in terms of their ability to process RNA substrates endonucleolytically, our results reveal that while some function directly in RNA degradation, a subset have roles downstream of initial endonucleolytic processing. We also demonstrate that the activities of SOX and Xrn1 are coupled for the efficient degradation of host mRNA transcripts.

## MATERIALS AND METHODS

### Protein expression and purification

The procedures used for SOX overexpression and purification (together with mutants) are described in Bagnéris *et al*. (4). The 6His-tagged K.*lactis* Xrn1 plasmid construct was a kind gift from L. Tong, Columbia University. Details for protein production and purification are described in Chang *et al*. (13).

### Oligonucleotides

Oligonucleotides used in the various studies are shown in Table 1.

**Table 1.** RNA and DNA sequences

Oligonucleotide	Sequence
GFP51	5΄-UACGGCAAGCUGACCCUGAAGUUCAUCUGCACCACCGGCAAGCUGCCCGUG-3΄FAM
HBB58	5΄-AGGUGAAGGCUCAUGGCAAGAAAGUGCUCGGUGCCUUUAGUGAUGGCCUGGCUCACCU-3΄FAM
GFP51-UCUCU	5΄-UACGGCAAGCUGACCCUCUCUUUCAUCUGCACCACCGGCAAGCUGCCCGUG-3΄FAM
GFP51-UGCAC	5΄-UACGGCAAGCUGACCCUGCACUUCAUCUGGUGCACCGGCAAGCUGCCCGUG-3΄FAM
K2-31	5΄-GAUCUGAGCCAUUGAAGCAAGCUUCCAGAUC-3΄FAM
K2-31A4	5΄-GAUCUGAGCCAUUGAAGCAAAAAGCUUCCAGAUC-3΄FAM
K2-31A9	5΄-GAUCUGAGCCAUUGAAGCAAAAAAAAAAAGCUUCCAGAUC-3΄FAM
UN51	5΄-GGCCAUCCUGUUUUUUUCCCUUUUUUUUUUUCUUUUUUUUUUUUUUUUUUU-3΄FAM
dsUN51	5΄-GGCCAUCCUGUUUUUUUCCCUUUUUUUUUUUCUUUUUUUUUUUUUUUUUUU-3΄FAM
	3΄-CCGGUAGGUCAAAAAAAGGGAAAAAAAAAAAGAAAAAAAAAAAAAAAAAAAAAA-5΄
dsDNA5΄P	5΄-pGGGGATCCTCCCAGTCGACC-3΄
	3΄FAM-CCCCTAGGAGGATCAGCTGG-5΄

All oligonucleotides were purchased from Eurogentec™. Those requiring further purification were subjected to gel extraction and processing using a ZR small-RNA™ PAGE recovery kit following the manufacturer's instructions in-house.

### RNase cleavage assay

The assay used to assess RNA degradation was based on that reported in Bagnéris *et al*. (4) which was adapted from the original protocol cited by Buisson *et al*. (3). While these conditions (1.35 μM SOX, 0.2 μM RNA, 25 mM Tris–HCl pH 9.0, 200 mM NaCl, 10 mM MgCl_2_ and 5 mM β-mercaptoethanol) supported efficient exonucleolytic processing of the unstructured 51mer (UN51) used by Buisson *et al.*, poor endonucleolytic cleavage was observed for GFP51 (Supplementary Figure S1A). It was therefore necessary to screen for more optimal conditions. To achieve this, 0.2 μM RNA was incubated with 1.35 μM SOX in 25 mM Tris-HCl (at pH intervals of 0.5 between 7.5 and 9.0), NaCl (varied between 50 and 200 mM), 10 mM MgCl_2_ and 5 mM β-mercaptoethanol (see Supplementary Figure S1B and C). Optimal cleavage was found to occur at pH 9.0 and 50 mM NaCl prompting all assays involving wild-type (WT) SOX alone (or mutants) to be performed in the original buffer reported for exonucleolytic processing, but with the NaCl concentration adjusted to 50 mM. For those involving Xrn1, that is inactive under these conditions, 1.35 μM WT SOX and/or 1.35 μM Xrn1 was incubated with 0.2 μM RNA in a buffer comprising 25 mM Tris–HCl pH 8.0, 100 mM NaCl, 1 mM DTT (dithiothreitol), 10 mM MgCl_2_, 100 μg/l bovine serum albumin (BSA). In this buffer, however, SOX is less active owing to the reduced pH and increased NaCl concentration (Supplementary Figure S1B and C). To compare the rates of turnover between oligonucleotides more quantitatively, time course assays were also conducted where samples were taken at 20-min intervals for 1 h. All other reactions were incubated for 1 h at 37°C and 7.5 μl of each mixture combined with 7.5 μl of Novex TBE (Tris-borate, EDTA)-urea sample buffer (Invitrogen) prior to loading onto 15% TBE-urea gels (Invitrogen or in-house) that were subsequently run in 1× TBE. The gels were then visualized using an FLA3000 transilluminator (FujiFilms) at excitation and emission wavelengths of 473 and 520 nm, respectively.

### Crystallization

E244S KSHV SOX at a concentration of 2 mg/ml was incubated at 4°C for 2–3  h with K2-31 using a protein:RNA ratio of 1:1.2 in a buffer comprising 32 mM Tris pH 8.3, 189 mM NaCl, 1.6 μg/l BSA and 10 mM DTT. The complex was then concentrated to 6  mg/ml (KSHV SOX) in a 0.5  ml Millipore^T^ 3  KDa cut-off centrifugal concentrator. A Mosquito^T^ robot was used to set up 200–300 nl drops in vapour diffusion, sitting drop crystallization trials using several commercially available screens. Both 1:1 and 1:2 ratios of complex to mother liquor solution were trialed. The best crystals were obtained using a 1:2 ratio after several days at 20°C from the condition 0.1 M magnesium acetate, 0.1 M MES, pH 6.5, 10% w/v PEG 10 000. These were subsequently harvested, cryoprotected in mother liquor solution containing 22% ethylene glycol and flash frozen in liquid nitrogen.

Data were collected on beamline I04 at DIAMOND on a single flash-frozen crystal to 3.3 Å. Images were processed and scaled using XDS and AIMLESS from the CCP4 suite (14). The structure was solved by Molecular Replacement using PHASER (15), manually re-built in COOT (16) and refined using AUTOBUSTER (17) incorporating cycles of TLS refinement. The final model comprises a single protein monomer, 18 nucleotides, 9 water molecules and has an *R*_free_ of 26.32%/*R*_work_ 20.77% (see Table 2). All stereochemical parameters are within the expected ranges for a structure at this resolution. The co-ordinates and structure factors have been deposited in the protein data bank under the accession code 5HSW.

**Table 2.** Crystallographic parameters

Space group	C2
Unit cell (a, b, c (Å))	198.2, 45.9, 67.6 α = γ = 90, β = 90.1
Resolution (Å)	44–3.3 (3.3–3.7)
Total number of reflections	30 812
Number of unique reflections	9 408
Redundancy	3.3 (3.3)
Completeness (%)	99.80 (99.8)
<*I*>/<σ(*I*)>	6.4 (1.8)
*R*_meas_ ^a^	0.19 (0.967)
Refinement	
Number of protein atoms	3232
Number of RNA atoms	380
Number of hetatoms	4
Number of solvent atoms	9
*R*_work_ ^b^/*R*_free_^c^ (%)	20.8/26.3
Estimated co-ordinate error based on *R*_free_ (Å)	0.527
Mean *B*-factor (Å^2^)	100.75
Deviations from ideal stereochemistry	
RMSD bonds (Å)	0.008
RMSD angles (°)	0.95
Wilson *B*-factor (Å^2^)	83.0
Ramachandran plot analysis^d^	
Most favored (%)	91.79
Additionally allowed (%)	6.76
Disallowed (%)	1.45

Values in parentheses are for the highest resolution shell (3.3–3.7 Å).

a *R*_meas_ = Σ((*N/N*-1))^1/2^(|*Ii* – <I>|)/Σ(<I>)), where the sum is calculated over all observations of a measured reflection (*Ii*), <I> is the mean intensity of all the measured observations (*Ii*) and N the total number of observations for each reflection.

b *R*_work_ = Σ (|F_obs_ – F_calc_|)/Σ (F_obs_), F_obs_ are the observed structure factor amplitudes, and F_calc_ those calculated from the model.

c *R*_free_ is equivalent to R_work_ but where 5% of the measured reflections have been excluded from refinement and set aside for cross-validation purposes.

d Ramachandran plot analysis was from molprobity (18).

### Fluorescence polarization anisotropy (FPA)

FPA was performed using 3΄FAM labeled oligonucleotides (see Table 1 and Results section). Details of the experimental setup and procedures used are as reported in Bagnéris *et al*. (4) with the exception that the data were processed in Graphpad PRISM6 and fitted using a single site binding equation.

### Yeast two hybrid screening

A previously validated library of human genes associated with mRNA degradation pathways was used to identify potential SOX partners where screens were performed as previously described (19). In brief, the SOX open reading frame was PCR (polymerase chain reaction) amplified from cDNA using the primers 5΄-ATG GAG GCC ACC CCC ACA CCC GCG GAC TTG-3΄ (forward), 5΄- GAT TTC TCC TAT CTA TCT GCA AAC GTC CCT CAC AGC CCG TAG-3΄ (reverse). The PCR product was then amplified using the primers 5΄- GAA TTC ACA AGT TTG TAC AAA AAA GCA GGC TGG ACC-3΄ (forward), 5΄- CGG GCT GTG AGG GAC GTT TGC AGA TAG ATA GGA GAA ATC CCA TTT GAT ATA TGG A-3΄ (reverse) for gap repair cloning into the pGBAD-B and pGACTBD-B vectors. These were then transformed into the yeast strain pJ69-4A and the red colonies picked from the resultant plate subjected to PCR to confirm that SOX had been successfully inserted as bait and prey plasmids. Autoreactive clones were removed from further mating. Positive clones were subsequently inoculated into the selective media SD-WHL+AT and SD-WAL for both bait and prey and incubated at 30°C for a minimum of 48 h. All positive colonies were further checked by plasmid sequencing and a functional β-galactosidase assay as described in Lehner *et al*. (19).

## RESULTS

### SOX-mediated endonucleolytic cleavage *in vitro* requires stem loop or bulge motifs

**Figure 1.**
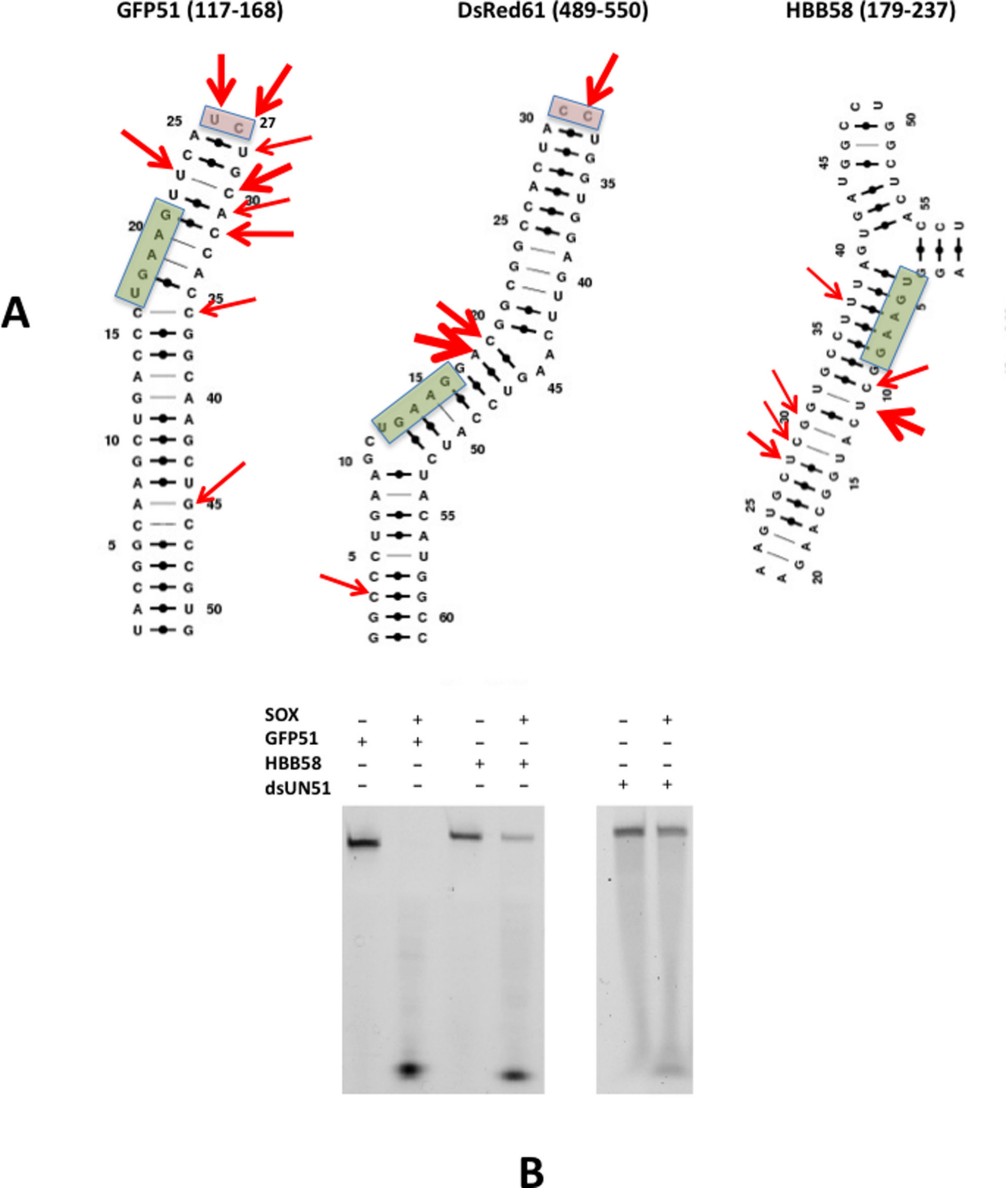
(**A**) The lowest energy secondary structure predictions for GFP51, DsRed61 and HBB58 generated by the MC-Fold | MC-Sym pipeline. Red arrows indicate cleavage sites reported by Covarrubias *et al*. (9) and green boxes highlight the UGAAG sequences. Thicker arrows represent sites that were targeted at higher frequency. Pink boxes highlight loops capped by two unpaired nucleotides reported to be cleavage sites. (**B**) RNase assays performed with GFP51, HBB58 (left) and dsUN51 (right).

In studies monitoring SOX-mediated turnover of GFP (green fluorescent protein), DsRed2 (red fluorescent protein) and β-globin (HBB) reporter mRNA in 293T cells (9), it was shown that although endonucleolytic cleavage yielded fragments of different lengths, the dominant sites all mapped to regions 3΄ to a UGAAG motif. More recently, a transcriptome-wide analysis has indicated that the sequence recognized is more likely to be degenerate, favoring di or tri-adenine within 6 nucleotides of the cleavage site, and that particular structures (i.e. stem loops) are the targets for mRNA degradation (11). Interestingly, analysis of the original GFP, DsRed2 and HBB transcripts revealed that additional cleavage sites (though less predominant) were also reported in regions devoid of any consensus sequence ((9) and Figure 1A). These observations prompted us to further investigate the three transcripts computationally. To facilitate this, mRNA sequences for the full-length GFP, DsRed2 and HBB transcripts were first subjected to *in silico* folding using default parameters in the program MFOLD (20). For GFP, 13 unique structures were obtained, 10 of which shared the same secondary structure in the vicinity of the UGAAG motif. These 10 structures corresponded to the lowest energy folds out of the 13 identified. Similar results were obtained with HBB, the only exception was that 14 solutions were obtained, 10 of which shared the same fold in the target UGAAG region. In contrast, DsRed2 yielded 39 structures where only 5 were found to be equivalent. Following the identification of stable substructures, the sequences were reduced to 201 nucleotides based on the lengths of transcripts reported to support cleavage (9). In all three, the substructures encompassing the targeted UGAAG sites remained unchanged. Cycles of *in silico* folding were then iteratively performed on sequences of incrementally reduced length until the minimal number of nucleotides could be identified which maintained the original substructures. These corresponded to 51 nucleotides for GFP (GFP51, nucleotides 117 to 168), 58 nucleotides for HBB (HBB58, nucleotides 179–237) and 61 nucleotides for DsRed2 (DsRed61, nucleotides 489–550). The resulting structures were also analyzed using the MC-Fold | MC-Sym pipeline (21) to produce secondary structure predictions in which non-canonical base pairs are included in energy calculations (the lowest energy predictions are shown in Figure 1A). Despite each being distinct, all form simple (GFP51) or more complex (HBB58 and DsRed61) structures consisting of one or more stem loops, where the cleavage sites are located within the loops themselves or adjacent to regions containing unpaired nucleotides. All have pyrimidines directly 3΄ to the major cleavage sites consistent with the recent studies of Gaglia *et al*. (11).

We next assessed whether the elements identified could be processed *in vitro*. To achieve this, the GFP51 and HBB58 sequences were synthesized as 3΄FAM substituted oligonucleotides (see Materials and Methods and Table 1) and analyzed using RNase assays (Figure 1B). Initial attempts using reaction buffers designed for unstructured substrates that are cleaved exonucleolytically (UN51, Supplementary Figure S1A and shorter oligonucleotides cited in Bagnéris *et al*. (11)) resulted in poor levels of cleavage. This necessitated buffer re-screening to establish the optimum conditions for degradation (see Materials and Methods together with Supplementary Figures S1A–C). Under these conditions, both GFP51 and HBB58 were found to be susceptible to degradation, in particular GFP51 (Figure 1B and Supplementary Figure S1D). Although discrete products resulting from endonucleolytic cleavage appear absent, this is largely due to rapid exonucleolytic processing following the initial incision given that fragments were frequently observed in assays of mutants defective in cleavage activity (see ‘Results’ section below).

To investigate whether the loops/bulges identified in GFP51 and HBB58 rendered substrates more readily processed than Watson-Crick base paired double-stranded RNA lacking these elements, assays were also performed with a double-stranded 51mer comprising UN51 and its complementary sequence (dsUN51, Table 1). dsUN51 was largely resistant to degradation (as shown in Figure 1B and the corresponding time course assays in Supplementary Figures S1D and E) in all buffers (data are only shown for those performed in the optimal endonucleolytic buffer) indicating that SOX-induced endonucleolytic processing is entirely dependent on the presence of loop or bulge elements within duplex regions of RNA substrates.

### Crystal structure of SOX bound to the KSHV pre-miRNA stem loop fragment K2-31

**Figure 2.**
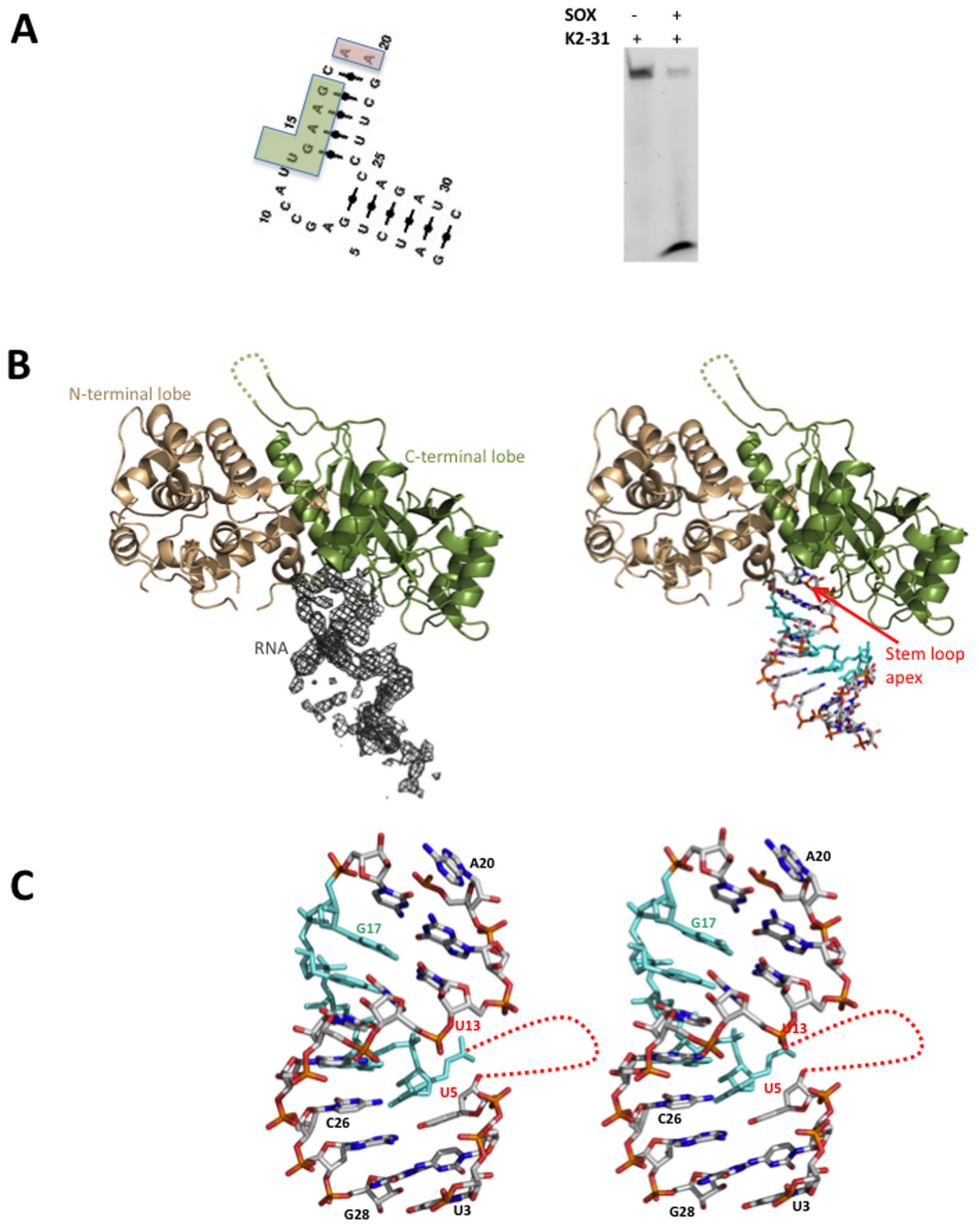
(**A**) Left. The lowest energy MC-Fold | MC-Sym secondary structure prediction for K2-31 (the UGAAG sequence is highlighted by a green box). Right. RNase assay performed with K2-31. (**B**) Cartoon representations of SOX (N and C-terminal lobes coloured wheat and dark green, respectively) together with (Left) the associated 2mFo-DFc omit map density (contoured at 1σ) for K2-31 bound in the ‘canyon’ and (right) with the RNA from the final model represented as sticks. The UGAAG sequence (U13-G17) is highlighted in cyan. Missing loops are included as dashed lines. (**C**) Stereoview showing the overall conformation of K2-31 (nucleotides U3-G28). Nucleotides 3΄ to U5 and 5΄ to U13 (red dotted line) constitute an internal 7 nucleotide loop that could not be modeled owing to disorder.

Attempts to co-crystallize SOX with either GFP51 or HBB58 were unsuccessful therefore, based on our computational studies, we sought to identify shorter RNA sequences more amenable to crystallographic studies. Since SOX degrades KSHV mRNA transcripts in addition to those of the host for the purposes of self-regulation (22), we focused on the KSHV transcriptome and in particular pre-miRNA's given their simple stem loop configurations. We were able to identify KSHV pre-miRNA K12-2 (K2), that also contains a similarly positioned UGAAG motif upstream of a bulge, as a likely target and using the same strategy to that outlined above, deduce that the structure could be maintained with only 31 nucleotides (K2-31, Figure 2A). This sequence was synthesized (incorporating a 3΄FAM group) and found to be degraded by SOX in RNase assays (Figure 2A and Supplementary Figure S1E). K2-31 was successfully co-crystallized with the SOX E244S catalytically inactive mutant (see Materials and Methods: Crystallization). Analysis of the crystal contacts revealed that key to lattice formation are RNA–RNA interactions (analogous to those involving DNA in the SOX-DNA complex 3POV, Supplementary Figure S2) mediated by symmetry-related molecules although only poor density could be observed for those involved in intermonomer contacts (nucleotides 1–3 and 29–31). These nucleotides were subsequently omitted from the deposited co-ordinates.

**Figure 3.**
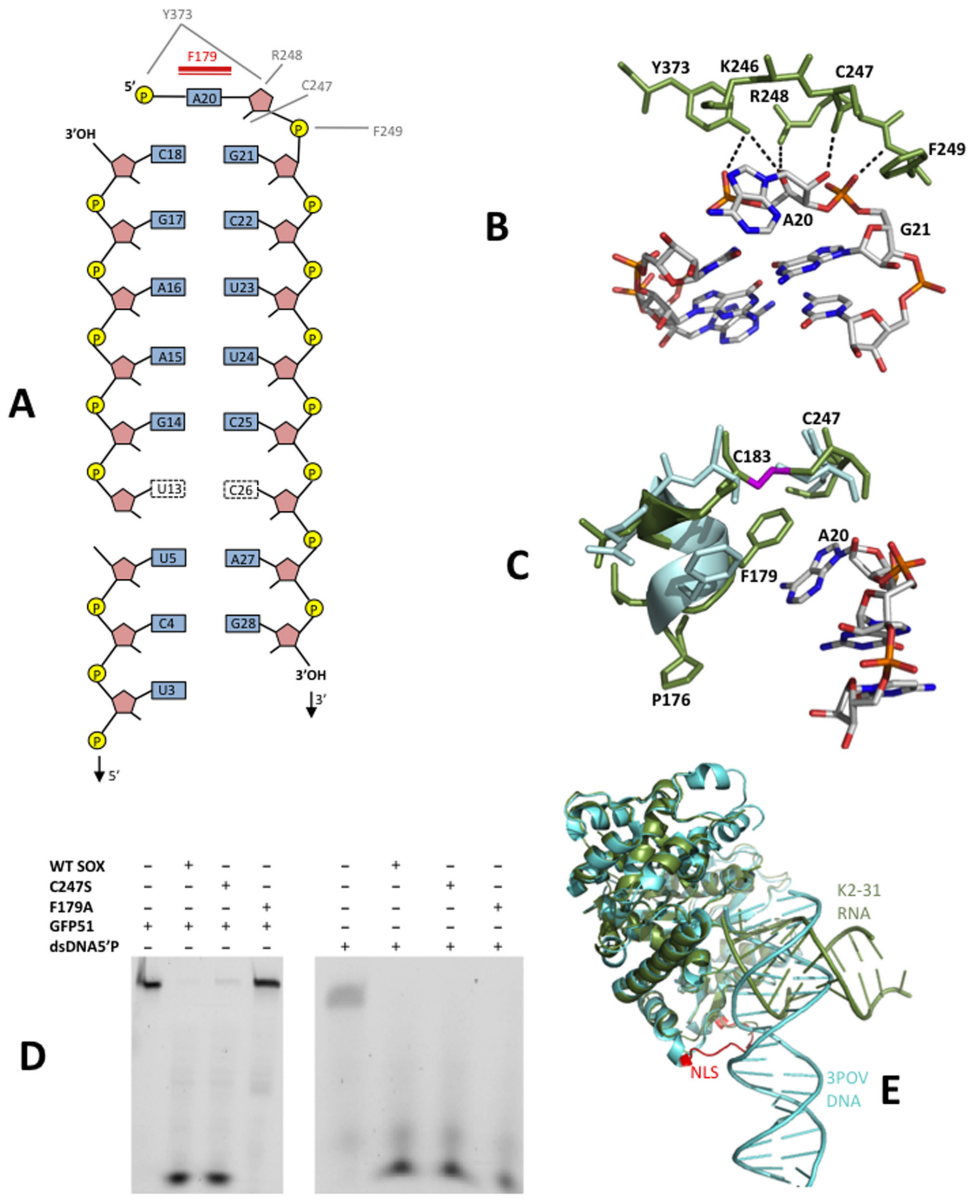
(**A**) Schematic diagram of the observed protein-RNA contacts in the SOX-K2-31 complex. Hydrogen bonding and π-stacking interactions are shown in grey and red, respectively. (**B**) The protein RNA contacts that function in loop recognition, central to which are those mediated by A20, G21 and residues K246, R248, F249 and Y373 within the catalytic region. (**C**) The protein–RNA interactions involving the bridge and the conformational re-arrangements observed in transitioning from the SOX–DNA complex, 3POV, (light blue) to SOX-K2-31 (dark green). The disulphide bond between C183 and C247, absent in 3POV, is shown in magenta. (**D**) RNase assays performed using the C247S and F179A mutants illustrating that while the C247S substitution is mildly defective, F179A is significantly impaired. (**E**) Superposition of 3POV and SOX-K2-31. Although the protein moieties in both complexes are near identical, an ∼90° rotation would be required to map the DNA co-ordinates onto those of K2-31 despite both interacting with the same catalytic residues. This is owing to K2-31 being stabilized exclusively in the loop region while additional contacts are evident in the DNA complex, notably, those involving the nuclear localization motif (NLS, red in 3POV).

Similar to the SOX-DNA structure (3POV), K2-31 is accommodated in the ‘canyon’ between the N and C-terminal lobes of SOX (Figure 2B) where the apex of the loop projects directly into the active site. Closer inspection revealed that K2-31 has a near continuous stem loop configuration, interrupted by the presence of a 7 nucleotide bulge (disordered in our structure) immediately 5΄ to the UGAAG motif. No density could be observed for A19 (Figure 2C). The overall K2-31 conformation is therefore congruent with the lowest energy secondary and tertiary structure MC-Fold | MC-sym predictions (Figure 2A) in which two nucleotides (A19 and A20) forming the loop apex are unpaired. The only deviation is that G6 (absent in our structure) appears not to form a Watson Crick base pair with C26 which alternatively stacks opposite U13 within the body of the stem duplex. Interestingly, the protein-RNA contacts stabilising the complex are narrowly distributed with most restricted to the catalytic region and the K2-31 loop (Figure 3A). In our structure, A20 and G21 at the apex are recognized by a series of hydrogen bonds involving Y373, R248 and F249 (Figure 3B and Supplementary Figure S3). While Nε1 of R248 donates a hydrogen bond to O4΄ of A20, the OH group of Y373 donates hydrogen bonds to both O4΄ and O1P. Also evident is a hydrogen bond donated by the 2΄OH group of A20 to the carbonyl oxygen of C247 as well as Van der Waals interactions contributed by the side chain of K246. Additionally, the peptide NH group of F249 donates a hydrogen bond to O1P of G21 (Figure 3B).

Further stabilization of K2-31 is derived from a stacking interaction between the adenine base of A20 and the aromatic side chain of F179 located in the bridge (Figure 3C). In order to achieve this, F179 moves ∼5 Å (based on Cα-Cα distances) relative to its position in the DNA complex. Surprisingly, this alternative configuration of F179 and the N-terminal end of the bridge is stabilized by a disulphide bond between the sulphydryl groups of C183 and C247 (Figure 3C). These interactions, absent in the reported SOX-DNA complex and apo structure, suggested that the bridge functions in the endonucleolytic processing of RNA substrates. To investigate this, F179A and C247S mutants were constructed and their ability to incise GFP51 assessed. Although C247S was only slightly impaired in cleavage activity compared to apo SOX, F179A was highly defective illustrating its important role in endonucleolytic processing (Figure 3D). These defects are RNA specific since both the C247S and F179A mutants readily degrade a DNA duplex substituted with a 5΄ phosphate (dsDNA5΄P).

Analysis of the SOX-K2-31 structure revealed that no specific interactions are observed between SOX and nucleotides 5΄ to A19. This apparent confinement of protein-RNA contacts to the K2-31 loop results in an overall binding configuration which contrasts markedly with that of the DNA duplex in 3POV, where there are additional contacts with the putative nuclear localization motif as well as those involving R248 and F249 detailed above (4), (Figure 3E). Consistent with this, the RNA stem loop and DNA duplex are mostly non-overlapping beyond the active site region, where an ∼90° rotation would be required to superpose K2-31 and the DNA co-ordinates. These differences are reflected in the burial of accessible surface area that constitute 240 and 480 Å^2^ for the SOX–K2-31 and SOX–DNA complexes, respectively.

**Figure 4.**
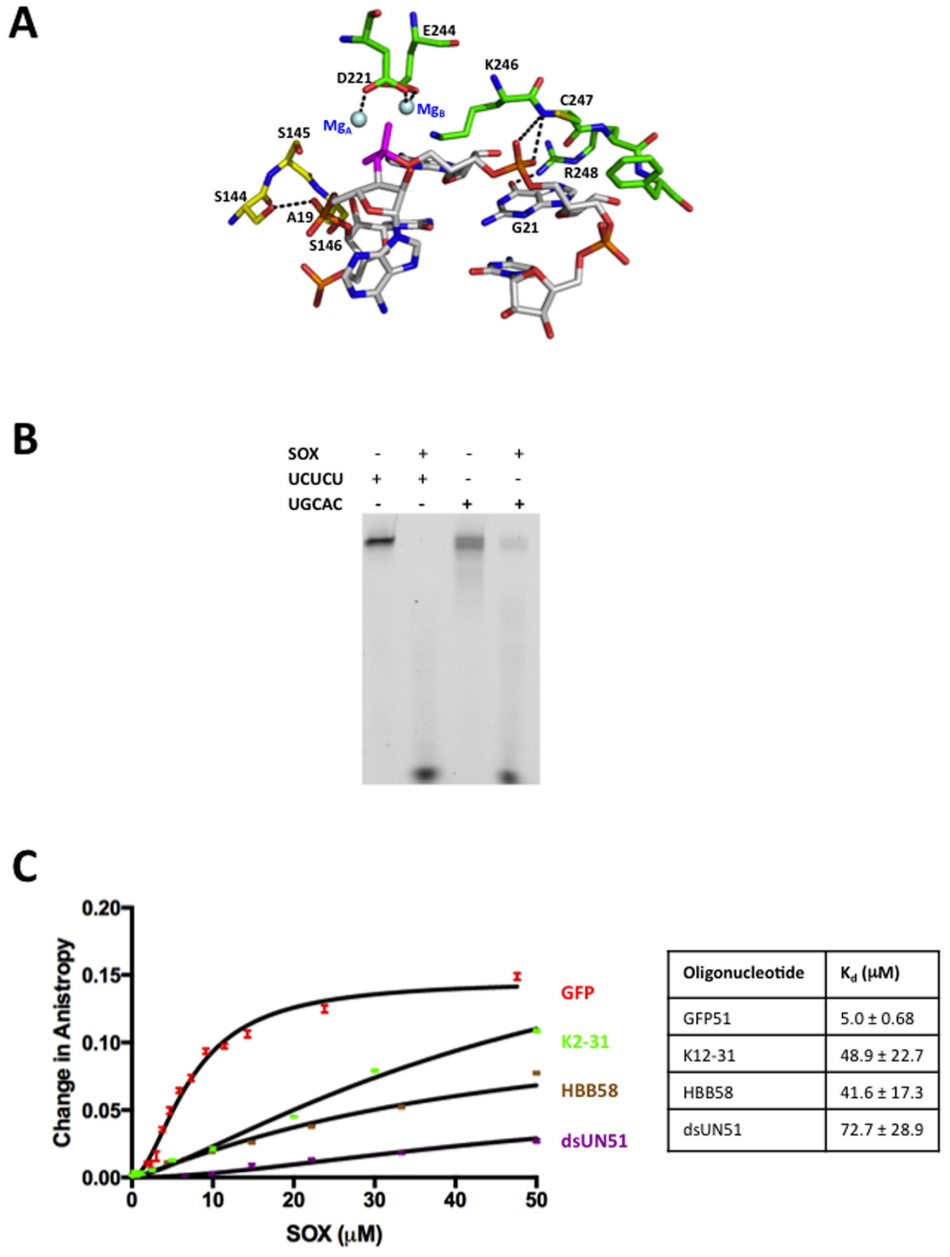
(**A**) Potential cleavage geometry of K2-31 based on an alignment with 3SM4. The most favorable configuration for catalysis is one in which the scissile phosphate (magenta) is located between magnesium ions A and B (Mg_A_ and Mg_B_, blue) where Mg_B_ is in the canonical site observed in 3SM4 and other type II restriction endonuclease-like enzymes but not 3POV. In this position, K2-31 is not only able to contact residues 247–249 (green) but also S144 reported to be essential for cleavage which forms a cluster with S145 and S146 that is conserved in a number of type II restriction-like enzymes (yellow). (**B**) RNase assays using the GFP51 template in which the UGAAG motif was substituted for UCUCU and UGCAC. (**C**) FPA assays in which WT SOX was titrated against GFP51, HBB58, K2-31 and dsUN51.

Despite use of the inactivated E244S SOX mutant, the absence of density for A19 and lack of a phosphate group in close proximity to the essential catalytic carboxylate D221, is indicative of K2-31 having undergone cleavage over the duration of the crystallization experiment to produce a product complex. Thus in order to investigate the most likely cleavage geometry for a stem loop substrate, an ‘intact loop’ model was generated for K2-31 by combining the co-ordinates for loop residues 18–22 from the lowest energy structure prediction of K12-2 (MC-Fold | Mc-Sym pipeline) with the remaining stem nucleotides from the crystal structure (Figure 4A). This model was subsequently docked onto the K2-31-SOX crystal structure. Although a configuration was observed in which the scissile phosphate could form a ligand with the non-canonical magnesium ion in 3POV, K2-31 would be distant from S144 that has also been shown to be essential for cleavage *in vivo* (9). S144, S145 and S146 form a cluster that is conserved in other type II restriction-like enzymes where it has been shown to function in 5΄ phosphate stabilization in the bacteriophage lambda DNA substrate complex (3SM4). Alternatively, aligning the scissile phosphate with that observed in 3SM4, which is approximately equidistant from Mg_A_ and Mg_B_ in their canonical positions, produces a configuration consistent with in-line attack based on the relative juxtapositions of the catalytic residues E244, D221 and K246 (Figure 4A). In this position, the phosphate group of A19 can be stabilized by S144 and important contacts between nucleotides 20–22 and residues 247–249 (though distinct to those in the product complex) still maintained. These findings point toward a geometry consistent with a canonical S_N_2 bi-metal nuclease mechanism for endonucleolytic cleavage.

### RNA turnover by SOX is consensus sequence independent

Inspection of the SOX-K2-31 structure reveals a complete absence of interactions between SOX and either the phophodiester backbone or bases of the UGAAG motif. To ascertain whether this might be a feature of the crystal structure or specific to K2-31, the role of the motif in cleavage was investigated by substituting the UGAAG sequence in GFP51 for UGCAC and UCUCU (GFP-UGCAC and GFP-UCUCU in Table 1). UGCAC was chosen to disrupt the AAG/A sequence reported to potentially influence targeting (11) while maintaining Watson Crick base pairing and UCUCU to replace all purines with pyrimidines. To rule out the possibility that the substitutions may have drastically altered the overall stem loop configuration, secondary structure predictions were generated using MC-Fold with those corresponding to the lowest energies revealing that the overall morphology of GFP51 remained largely unchanged (Supplementary Figure S4), although an additional two nucleotide bulge was observed for the UGCAC substitution three nucleotides 5΄ to the first major cleavage site. RNase assays revealed that substitution of the UGAAG motif for either UGCAC or UCUCU failed to appreciably impair SOX-mediated degradation suggesting that cleavage *in vitro* is not significantly dependent on conservation of a consensus sequence (Figure 4B). Although it could be argued that this apparent lack of impairment could be attributable to the incubation times and quantities of SOX used, a time course experiment was also conducted involving GFP51 and GFP51-UCUCU (Supplementary Figure S5). This revealed that turnover of GFP51-UCUCU by SOX is as efficient as that observed for GFP51 confirming that the UGAAG motif is not the key driver for transcript processing.

**Figure 5.**
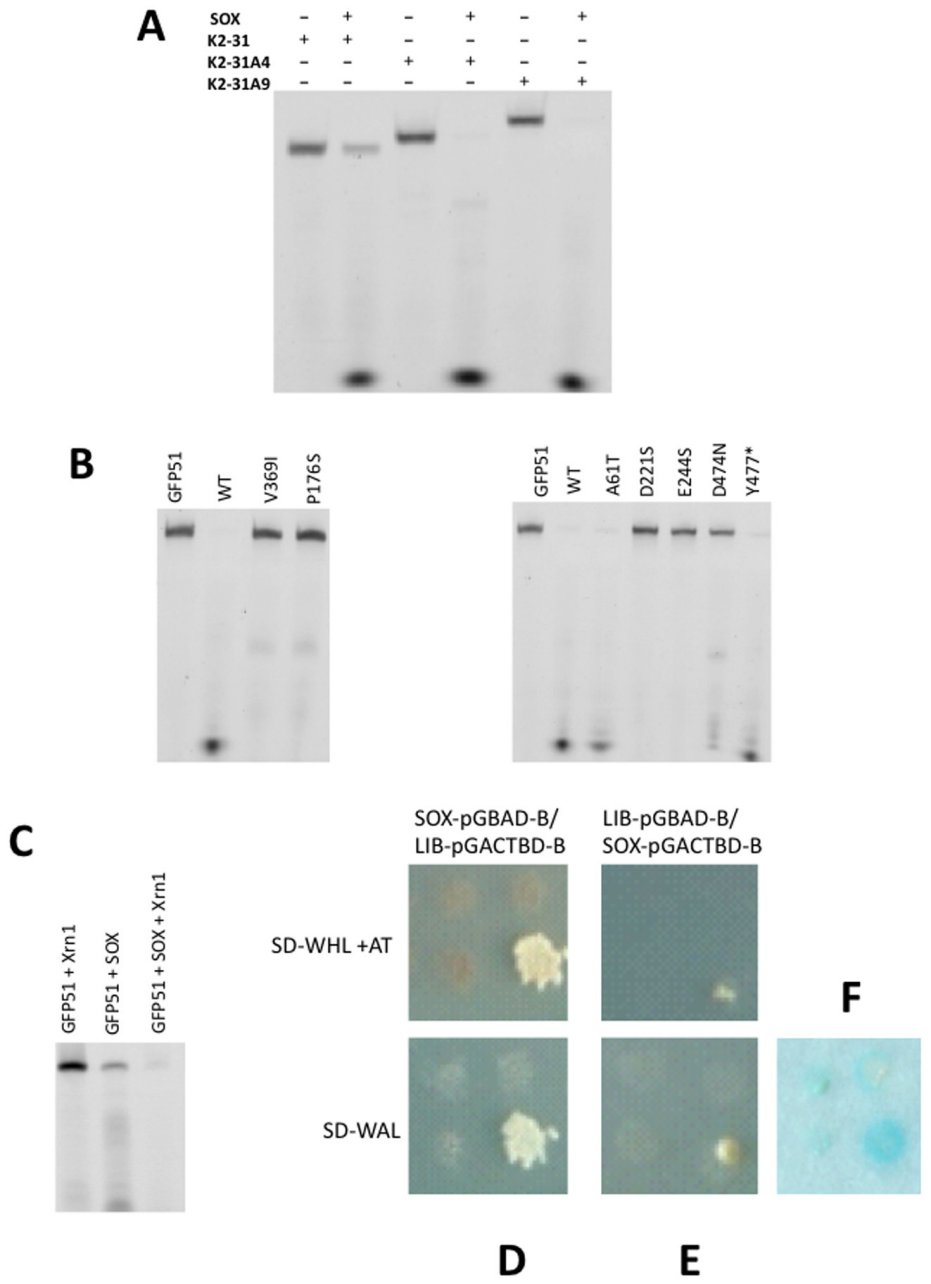
(**A**) RNase assays performed using the K2-31 variants K2-31A4 and K2-31A9 where three and nine adenines were introduced to the K2-31 loop apex, respectively. (**B**) RNase assays of WT SOX and the HSO mutants A61T, P176S, V369I, D474N and Y477 stop using GFP51. The D221S and E244S catalytic mutants were included as negative controls. (**C**) RNase assays performed using WT SOX, GFP51 and Xrn1 illustrating enhanced cleavage when SOX and Xrn1 are combined. (**D**) A yeast two hybrid screen using a selective library involving human proteins exclusively involved in mRNA degradation illustrating an interaction between SOX and Xrn1. Cells containing SOX as bait (SOX-pGBAD-B) and the library proteins as prey (Lib-pGACTBD-B) were grown in the selective media SD-WHL + AT (top) and SD-WAL (bottom), respectively. (**E**) As for (D), but where the library was used as bait (Lib-pGBAD-B) and SOX prey (SOX- pGACTBD-B). Colonies were only observed for those containing SOX and Xrn1 that were verified by sequencing. (**F**) β-galactosidase assay of clones identified as positive and negative for SOX and Xrn1. The most intense blue plaque corresponds to those positive for SOX and Xrn1.

Given that endonucleolytic processing appears to be independent of a consensus sequence while loop/bulge elements are required for cleavage, we next investigated the affinity of SOX for GFP51, HBB58, K2-31 and dsUN51 to establish whether such features are preferentially bound (Figure 4C). Of the oligonucleotides tested, SOX had the highest affinity for GFP51 with a *K*_d_ of 5 μM compared to 42 and 49 μM for HBB58 and K2-31, respectively. The highest *K*_d_ was observed for dsUN51 at ∼73 μM. These results demonstrate a clear binding preference for simple stem loops over bulges. Additionally, expanding the loop in K2-31 to incorporate three and nine additional adenines failed to negatively impact on turnover (Figure 5A) indicating that although stem loops are required for optimal endonucleolytic processing, there are no limitations on size. This lack of apparent specificity would account for the disparate nature of the RNA targets identified *in vivo* reported by Gaglia *et al*. (11).

### The HSO mutants have distinct roles in RNA processing

Having probed the nature of the SOX RNA targets, we next deduced whether the apparent failure of the HSO mutants to impair unstructured single-stranded RNA processing reported, could be attributed to the absence of stem loops and bulges in such oligonucleotides. They were therefore re-evaluated in terms of their capacity to degrade GFP51. The mutants tested constitute A61T (within the N-terminal lobe), P176S (located in the bridge region), V369I (at the center of the C-terminal lobe), D474N and Y477Stop (Y477*) at the C-terminus of SOX (Supplementary Figure S6). T24I (an additional HSO mutant) could not be assessed owing to insolubility as previously reported (4)). RNase assays revealed that the mutants can be largely subdivided into two categories: those that are profoundly defective in GFP51 cleavage activity and those that have a minor-to-moderate defect (Figure 5B). The mutations P176S and V369I almost completely abolish processing in agreement with *in vivo* studies where they virtually abrogate HSO (10). Given that P176 is located within the ‘bridge’, it can be envisaged that the P176S mutation has the potential to disrupt the conformational re-arrangements required for F179 to engage with RNA targets in order to confer substrate/product stabilization based on our earlier results. Similarly, V369 is located in a small hydrophobic recess, where its replacement with isoleucine would result in the re-positioning of residues directly adjacent to the catalytic region, in particular those in close proximity to A20. By contrast, D474N and Y477* have a moderate to minor effect on RNase activity. Their lower impact can be explained by their locations at the C-terminus of SOX (>30 Å from the catalytic region). Similarly, the A61T mutant, located ∼29 Å from the active site, appears to have wild-type activity. These results suggest that the far C-terminus of SOX and A61 do not have a role in the initial endonucleotlytic cleavage of mRNA transcripts.

### The RNase activities of SOX and Xrn1 are coupled

Although it has been shown that Xrn1 is essential for HSO (9), it was unclear whether the products of SOX cleavage generate Xrn1 substrates. To assess this, RNase assays involving SOX using optimized conditions for Xrn1 activity (sub-optimal for SOX cleavage, see Materials and Methods), were conducted in the presence and absence of K. *lactis* Xrn1 that has 54% sequence identity to the human homolog in the catalytic domain. While Xrn1 in isolation is incapable of degrading GFP51 (Figure 5C), enhanced degradation is observed when it is combined with SOX. Our results therefore confirm that the products of SOX cleavage are substrates for Xrn1 and that additional factors are unlikely to be essential at these initial stages of target processing. Moreover, the increased rate of turnover indicates that the activities of SOX and Xrn1 act in concert to facilitate rapid processing. In agreement with this, yeast two hybrid studies involving SOX as both bait and prey using a library composed of proteins exclusively involved in human mRNA degradation pathways reveal that Xrn1 and SOX physically interact (Figure 5D–F).

## DISCUSSION

Pivotal to a detailed understanding of KSHV mediated HSO is knowledge of the targets degraded and in particular, the nature of their interaction with the HSO nuclease SOX. Although recent *in vivo* studies have identified RNA stem loops as substrates and a potential requirement for sequence dependency, little could be ascertained about their structure. This problem derived from the seemingly diverse nature of the elements targeted given not only the variations in their sequences, but also the fact that incision sites have been identified in both loop and stem segments of structured mRNA transcripts. Despite this, cleavage appears to be restricted to regions within, or flanked by, unpaired nucleotides (11). In efforts to address these important issues, we first generated *in silico* models of three transcripts shown to be SOX targets and were able to identify the minimal sequences required to maintain the folds of these elements. We were able to show *in vitro* that they are incised by SOX while Watson Crick base paired double-stranded RNA of a comparable length is resistant to processing.

Analysis of the identified elements revealed that all three transcripts incorporate single stem loops or bulge/loop elements within or adjacent to the reported cleavage sites. Based on these common features, we were able to both identify and verify KSHV pre-miRNA K12-2 (K2) as a potential target using a 31mer fragment (K2-31). K2, an RNA polymerase II transcript that is expressed from latency, has been reported to have an important role in influencing cytoskeletal organization through suppressing the expression of high molecular weight tropomyosin 1 splice variants (HMW-TPM1) (12). Although how K2 functions remains unclear, the suppression of HMW-TPM1 has been directly linked to downregulated anoikis. This has been associated with KSHV infection as well as metastasis in several non-viral cancers where their expression is near abolished. An important contributor to viral trafficking could thus be the overproduction of K2 and it is interesting to speculate that SOX may have a role in the maturation of its pre-miRNA. In support of this, it has been established that SOX-mediated degradation of the KSHV transcriptome has an important role in the production of viable progeny (22) and that pre-miRNAs containing short loops are often poor substrates for dicer, the host nuclease that is required for miRNA maturation via the canonical pathway (23) (although other mechanisms have been identified). It has also been reported that KSHV miRNA's K12-1 to K12-8 are highly expressed during the lytic phase in the PELs cell line BC3 (24). Intriguingly, the 5΄-3΄ degradation of pre-miRNA K2 by SOX/Xrn1, initiated by endonucleotic cleavage at the loop apex, would result only in destruction of the passenger strand (Supplementary Figure S7) leaving the guide strand intact. This could potentially be passed to the RISC complex for engagement with its target. Our findings thus present the possibility that KSHV has the potential to not only subvert the host RNA degradation machinery but also machineries involved in miRNA targeting.

In addition to identifying K2 as a target *in vitro*, we were able to successfully crystallize a product complex involving a K2 31mer fragment. This structure has established the structural basis for mRNA transcript recognition and processing. Key to its formation is loop recognition in which one of the two unpaired adenines at the apex, together with the following 3΄ guanine, contact residues that collectively form part of the catalytic region (namely K246, R248 and F249). As well as contacts involving residues key to catalysis, F179 further stabilizes the SOX–K2-31 complex following re-modeling of the bridge that appeared to involve formation of a disulphide bond between the sulphydryl groups of C183 and C247. Our mutagenesis studies have shown that while C247 has a minor role, F179 is essential for RNA but not DNA processing confirming the bridge as an important factor in substrate/product recognition where it directly functions in RNA binding as previously hypothesized (7). Interestingly, comparison of the bridge regions in BGLF5 and SOX reveals several differences, the most significant of which are re-arrangements in the P158 region (equivalent to P176 in KSHV SOX) in response to the deletion of two upstream amino acid residues and the substitution of A174 for glycine. These differences may alter how RNA substrates are bound and processed potentially resulting in distinct preferential cleavage sites to those observed for KSHV SOX as reported in Covarrubias *et al*. (25).

Despite K2-31 in our structure having undergone processing, we have been able to model a potential substrate complex arising from alignments involving a ‘loop intact’ model and the co-ordinates from 3SM4 in keeping with a bi-metal nuclease mechanism. Based on the positions of the conserved magnesium ions and sites known to be essential for binding (i.e. S144 and residues 247–249) and cleavage, the scissile phosphate is more likely to interact with MgB in its canonical position with respect to other type II restriction endonuclease-like enzymes.

Surprisingly, the SOX–K2-31 complex showed no evidence of protein–RNA contacts beyond the catalytic region including the UGAAG motif. This apparent lack of dependency on a consensus sequence for transcript recognition was supported by our RNase assays in which its substitution for UCUCU or UGCAC in GFP51 failed to attenuate cleavage. Also noteworthy is the absence of this motif in the vicinity of the loop structure of DsRed61 (Figure 1A) reported as an incision target (9). Our studies additionally show that SOX appears to have a binding preference for simple stem loops over substrates comprising those that are more complex or bulges, consistent with the higher rates of turnover associated with GFP51 and UCUCU. There also appear to be no restrictions on loop size based on assays involving the K2-31 loop variants.

Although our results point to architecture and not sequence being the major pre-requisite for cleavage, the preference for an adenine rich degenerate consensus sequence 5΄ to cleavage sites *in vivo* was reported by Gaglia *et al*. (11). This could be explained by the propensity of adenine to induce bends/bulges into RNA duplexes when unpaired. In keeping with this, it has been shown that the insertion of unpaired adenine stretches has a greater capacity to distort RNA duplexes than pyrimidines (26,27). Although purines have been shown to be under-represented directly 3΄ to cleavage sites, the origins of this preference remain unclear based on our current data. This poor representation may arise from the potential of adenine and in particular guanine to adopt conformations that inhibit substrate binding or product dissociation in this position as a result of interactions with non-catalytic residues. These hypotheses, however, have yet to be investigated.

The identification of substrates susceptible to endonucleolytic cleavage led us to re-evaluate the HSO mutants to ascertain whether their activities might be impaired relative to the unstructured substrates originally trialed. Interestingly, a range of results were obtained in RNase assays involving GFP51 that largely mirrored those reported *in vivo*. Perhaps not surprisingly, the P176S and V369I mutants are highly defective in RNase activity most likely as a consequence of their roles in substrate/product stabilization and/or proximity to key residues forming the active site. In contrast, D474N showed attenuation while A61T and Y477* had near wild-type activity. Based on the crystal structure, these residues are distant from the catalytic site and are thus unlikely to have a direct impact on RNA recognition. We were also able to ascertain that the products of SOX cleavage are indeed substrates for Xrn1 and that the enhanced degradation of transcripts evident when both are combined is suggestive of their exoribonuclease activities being coupled, most likely through a physical interaction as suggested by the results of yeast two hybrid analysis. On aggregate, our results are consistent with a dynamic system for KSHV mediated mRNA degradation in line with *in vivo* studies.

## Supplementary Material

Supplementary Data
