# Supplementary material for: KSHV SOX mediated host shutoff: the molecular mechanism underlying mRNA transcript processing
Source: Nucleic Acids Res. 2017 Jan 28;45(8):4756–67. doi: 10.1093/nar/gkw1340 (PMC5416870; doi:10.1093/nar/gkw1340)
Supplement: Supplementary Data [file gkw1340_Supplementary_Data.zip › nar-00294-v-2016-File002.docx]

**Supplemental Data**

**
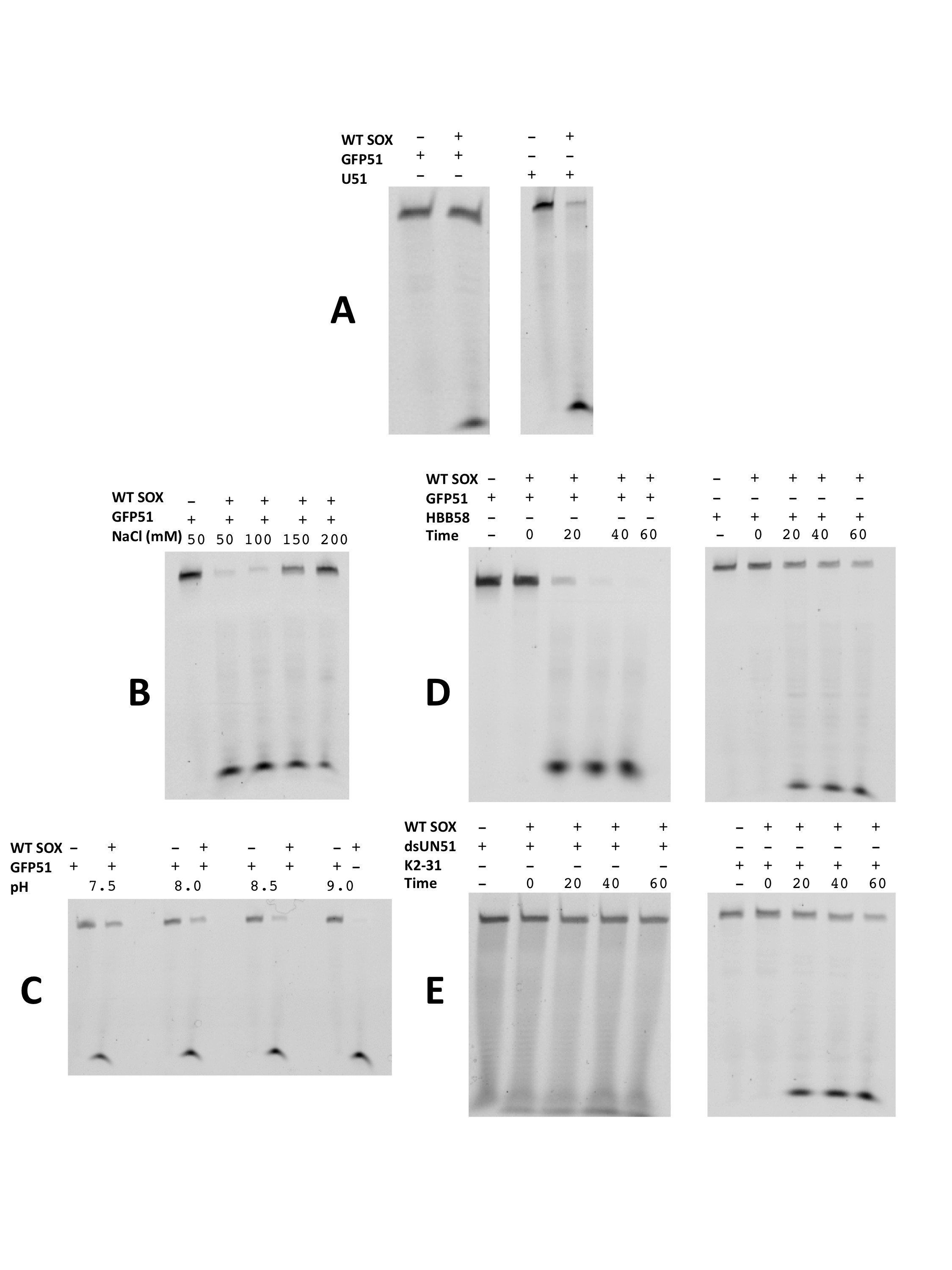
**

**Figure1. (A).** SOX RNase assays for GFP51 (left) and U51 (right) performed using the buffer that supports efficient exonucleolytic cleavage of U51. **(B)** Assays in which the NaCl concentration was varied to establish optimum conditions for GFP51 cleavage. **(C)** Similar to **(B)** but where pH only was screened (NaCl concentration fixed at 50 mM). Time course assays to more quantitatively compare the rates of SOX mediated turnover for GFP51 and HBB58 **(D)** and dsUN51, K2-31 **(E)**.

**
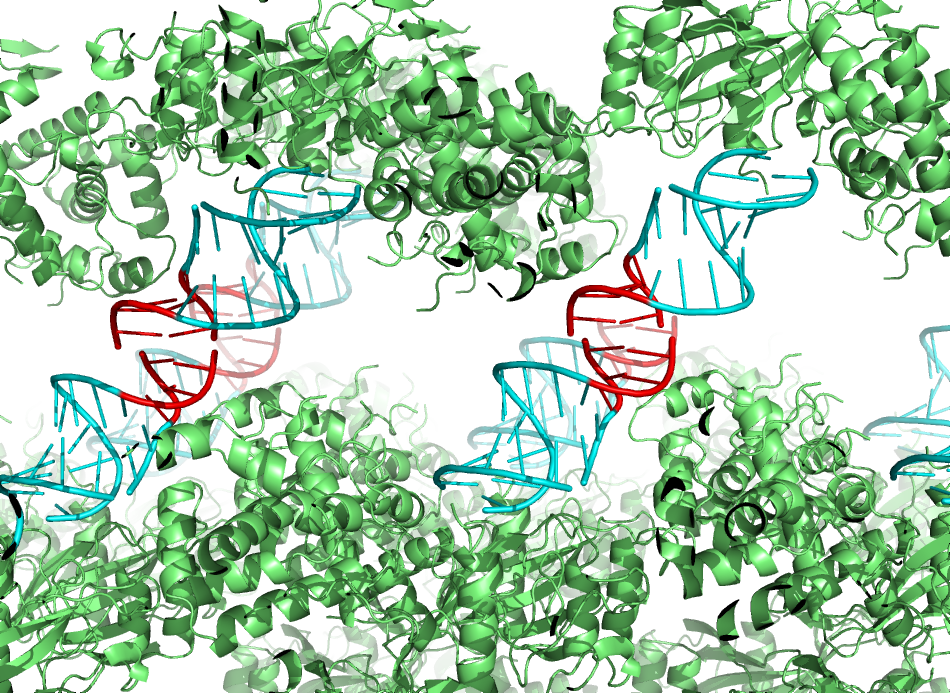
**

**Figure 2.** Crystal packing diagram illustrating that key to formation of the lattice are RNA-RNA interactions (cyan). Nucleotides highlighted in red forming the interface between symmetry related monomers are poorly ordered. Protomers are coloured green.


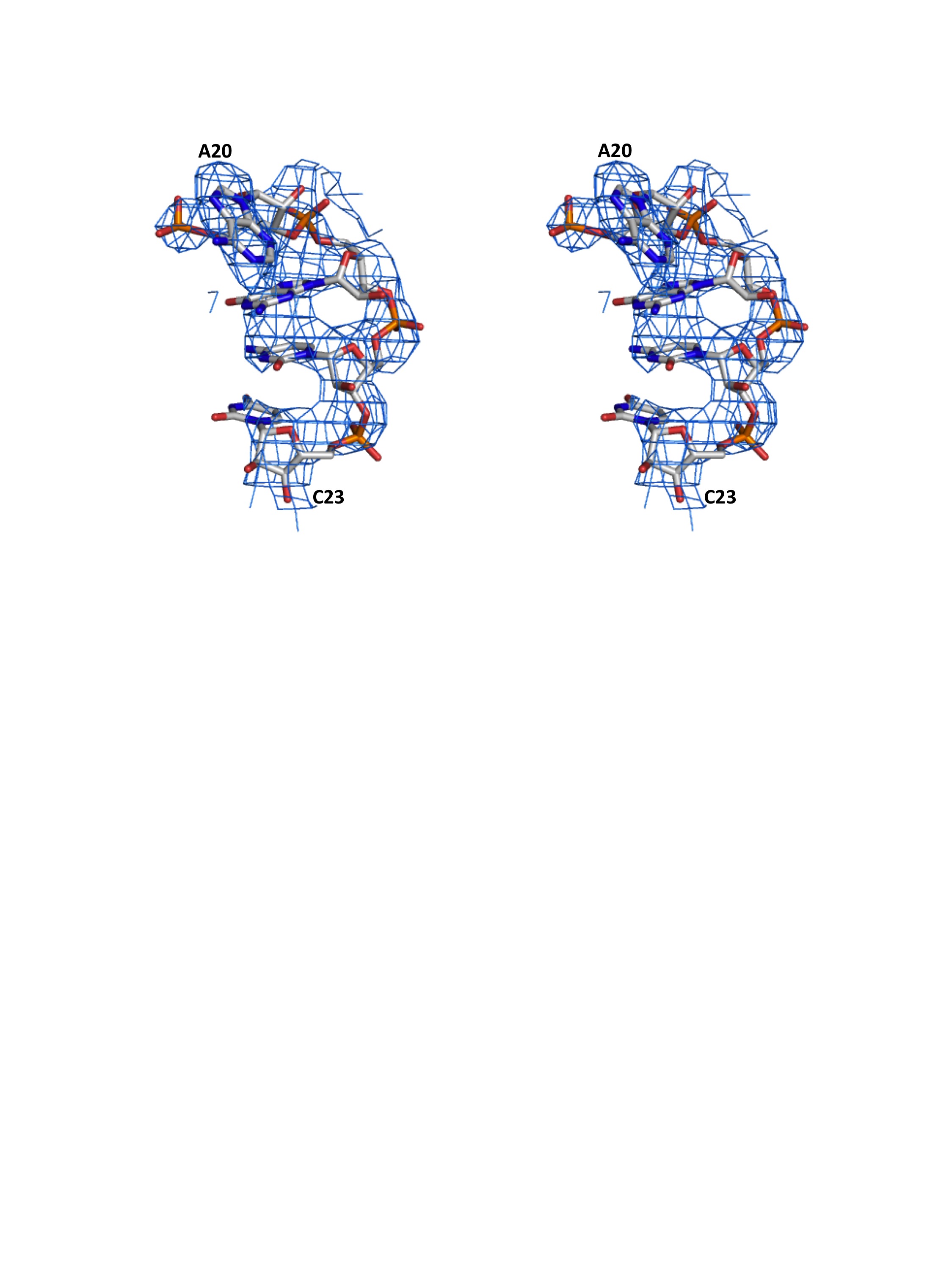


**Figure 3**. 2mFo-DFc omit map density (blue) contoured at 1σ, together with the

co-ordinates for nucleotides 20 to 23 in the final model.

**
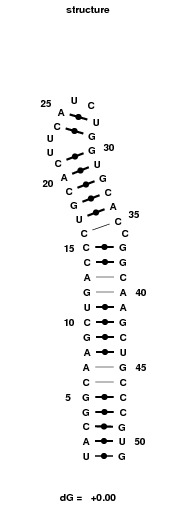

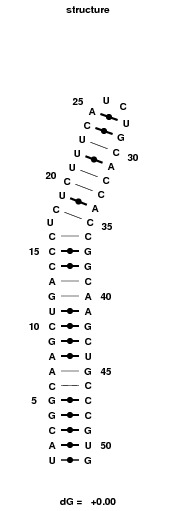
**

**Figure 4.** The MC-Fold | MC-Sym lowest energy secondary structure predictions for GFP51 where the UGAAG motif has been substituted for (left) UGCAC and (right) UCUCU.


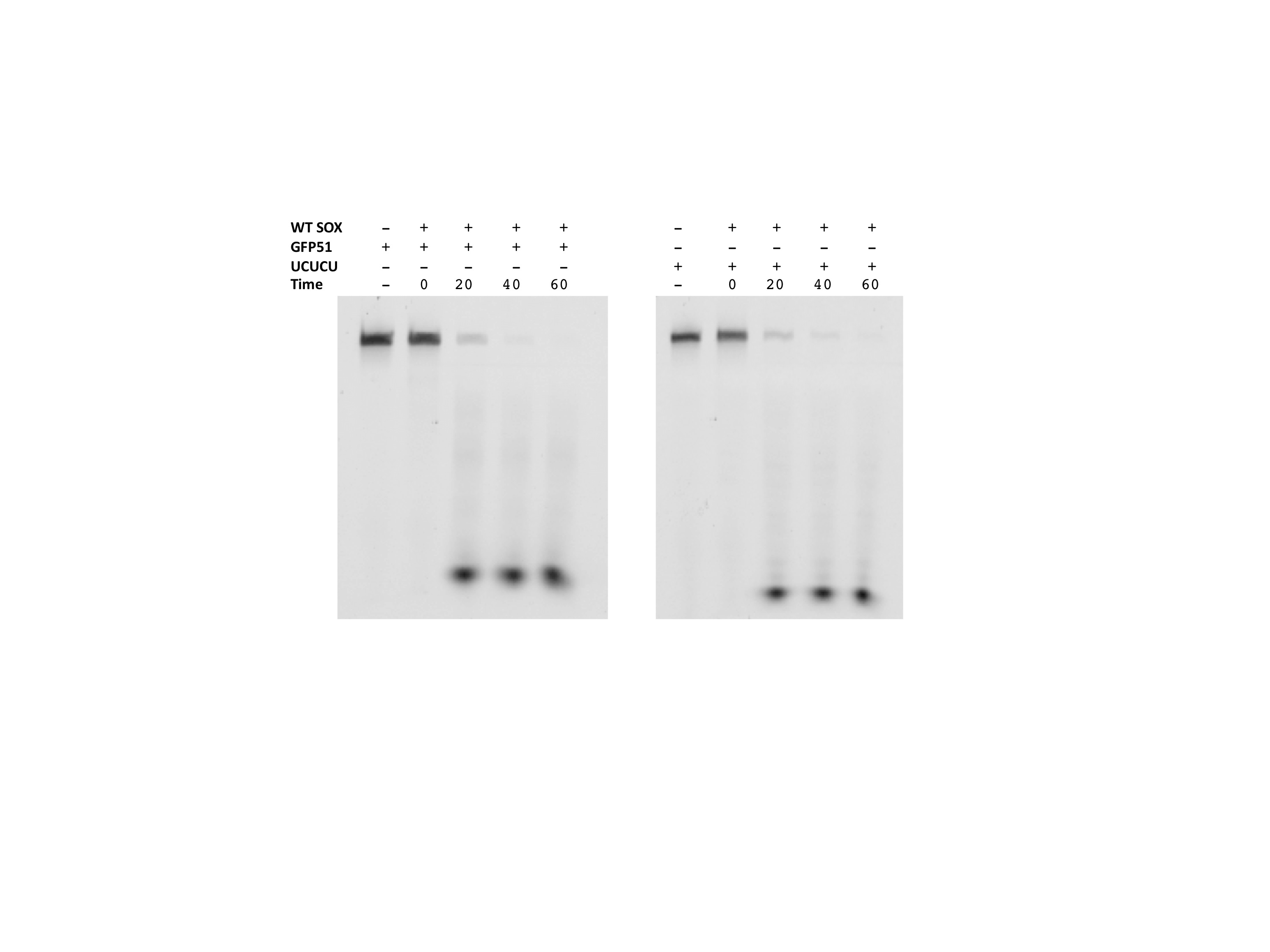


**Figure 5.** RNase assay time course experiments to qualitatively compare the rates of turnover for GFP51-UCUCU and GFP51 in the presence of SOX.


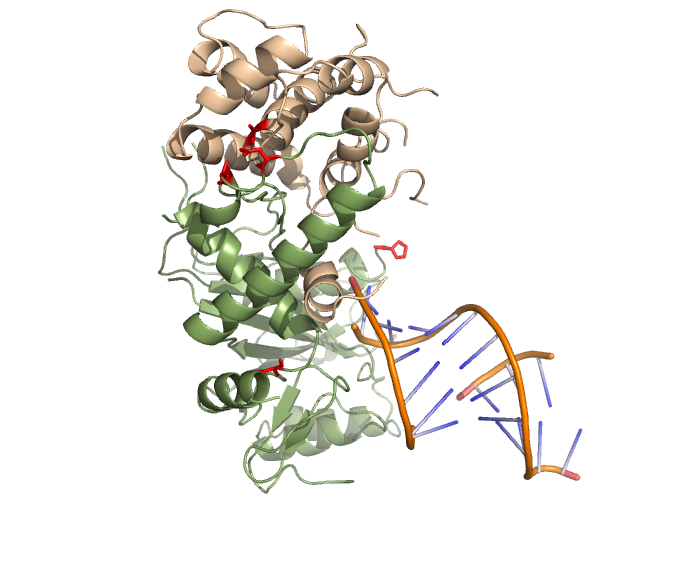


**V369**

**P176**

**D474**

**Y477**

**A61**

**Figure 6.** Protein cartoon of the SOX-K2-31 complex with the residues implicated in HSO highlighted in red.

**
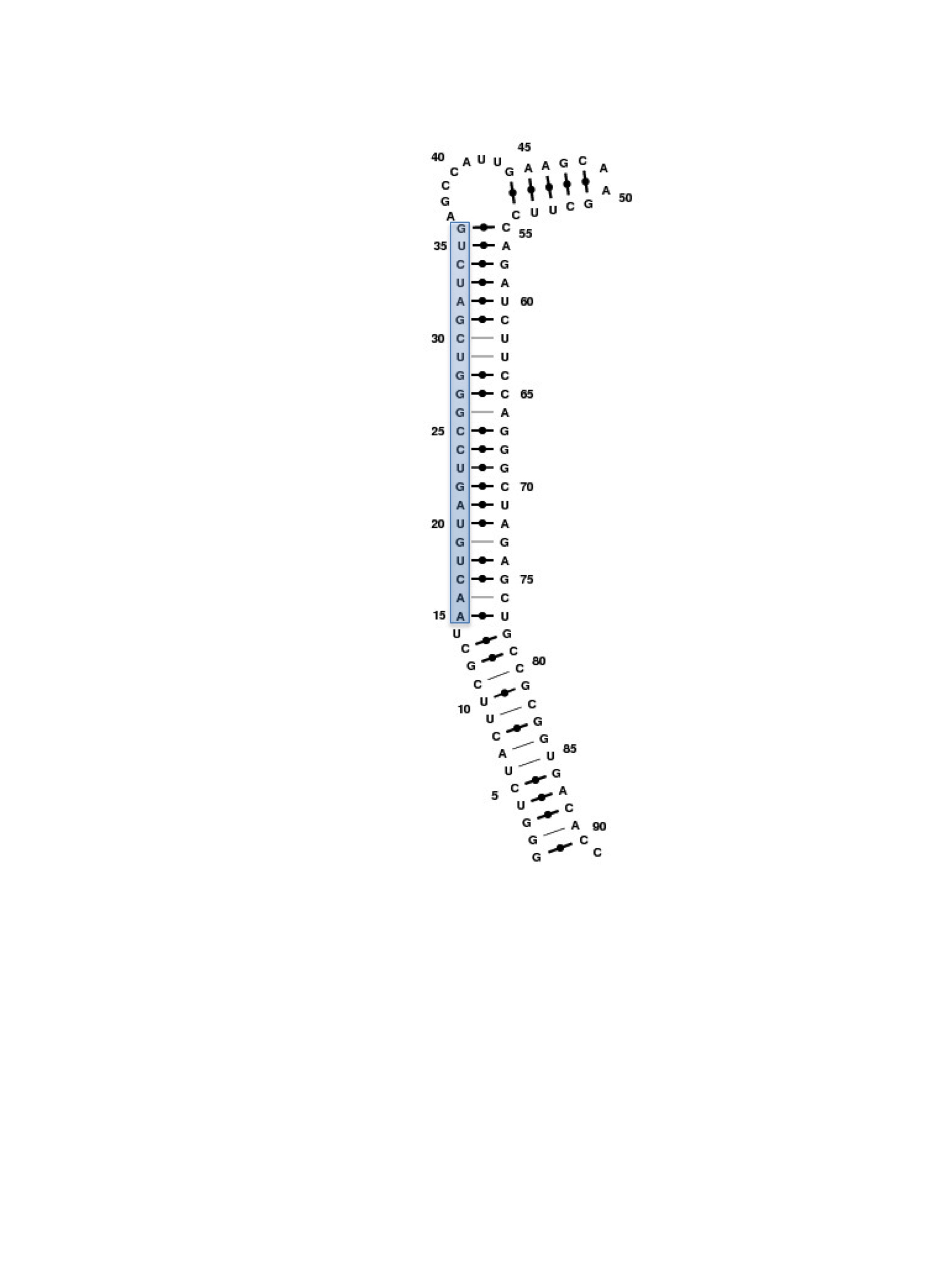
**

**Figure 7.** MC-Fold | MC-Sym lowest energy secondary structure prediction for pri-miRNA K12-2 (K2) with the guide strand highlighted in blue
